# Combined Metabarcoding and Multi-locus approach for Genetic characterization of *Colletotrichum* species associated with common walnut (*Juglans regia*) anthracnose in France

**DOI:** 10.1038/s41598-018-29027-z

**Published:** 2018-07-17

**Authors:** Daniele Da Lio, José F. Cobo-Díaz, Cyrielle Masson, Morgane Chalopin, Djiby Kebe, Michel Giraud, Agnes Verhaeghe, Patrice Nodet, Sabrina Sarrocco, Gaetan Le Floch, Riccardo Baroncelli

**Affiliations:** 1 Laboratoire Universitaire de Biodiversité et Ecologie Microbienne, IBSAM, ESIAB, Université de Brest, EA 3882, Technopôle Brest-Iroise, 29280 Plouzané, France; 2 Station Expérimentale Nucicole Rhône-Alpes, 385 A Route de St Marcellin, 38160 Chatte, France; 3 Centre Technique Interprofessionnel des Fruits et Légumes, Centre de Lanxade, 28 route des Nébouts, 24130 Prigonrieux, France; 4 CTIFL/SENuRA, 385 A Route de St Marcellin, 38160 Chatte, France; 5 Dipartimento di Scienze Agrarie, Alimentari e Agro-ambientali, Università di Pisa, Via del Borghetto 80, 56124 Pisa, Italy; 6 Present Address: Dipartimento di Scienze Agrarie, Alimentari e Agro-ambientali, Università di Pisa, Via del Borghetto 80, 56124 Pisa, Italy

## Abstract

*Juglans regia* (walnut) is a species belonging to the family *Juglandaceae*. Broadly spread in diverse temperate and subtropical regions, walnut is primarily cultivated for its nuts. In France, *Colletotrichum* sp. on walnut was detected for the first time in 2007; in 2011 the disease led to 50–70% losses in nut production. A combined approach of metabarcoding analysis and multi-locus genetic characterization of isolated strains has been used for taxonomic designation and to study the genetic variability of this pathogen in France. Evidence indicates that four *Colletotrichum* species are associated with walnut in France: 3 belong to the *C*. *acutatum* species complex and 1 to the *C*. *gloeosporioides* species complex. Results also show that *C*. *godetiae* is the most abundant species followed by *C*. *fioriniae*; while *C*. *nymphaeae* and another *Colletotrichum* sp. belonging to the *C*. *gloeosporioides* complex are found rarely. Representative isolates of detected species were also used to confirm pathogenicity on walnut fruits. The results show a high variability of lesion’s dimensions among isolates tested. This study highlights the genetic and pathogenic heterogeneity of *Colletotrichum* species associated with walnut anthracnose in France providing useful information for targeted treatments or selection of resistant cultivars, in order to better control the disease.

## Introduction

The English/Persian walnut (*Juglans regia* L., 1753), or common walnut, is a species that is native to Central Asia and belongs to the Juglandaceae family. The genus *Juglans* includes approximately 21 species; all species produce nuts but only *Juglans regia* is extensively cultivated for commercial production^1^. The common walnut is a tree broadly spread in diverse temperate and subtropical regions of North and South America, Asia, Australia, New Zealand, South Africa and Europe, where it grows widely or semi-cultivated. In Europe, common walnut was most likely introduced from Iran and eastern Turkey by Greek commerce a thousand years ago^2^. Common walnut is primarily cultivated for its nuts, which are harvested from wild stands, backyard gardens or commercial orchards. Nuts are collected for home consumption or sold on the market for their nutritional values and their high polyunsaturated fats content, including omega-3, consumed either as a snack or in baked foods. Furthermore, walnut trees are utilized for their high quality wood to make a wide array of products^3^. The total world production of *J*. *regia* is estimated to be about 3.4 million tonnes; China is the world’s largest producer of walnuts with a total production of about 1.7 million tonnes^4^. In 2014, European Union produced about 169,621 tonnes of walnuts with France the largest producer with about 34,767 tonnes of walnuts yielded, followed by Romania (31,514 tonnes) and Greece (22,310 tonnes)^4^. In France walnut cultivation occupies an area of about 19,712 ha^4^; orchards are the main production sites whereas harvest on isolated trees has strongly decreased in the last decades. In France, the establishment of new orchards, mainly localised in two large areas, balanced this reduction: South-East (Auvergne-Rhône-Alpes region) and the South-West (mainly Dordogne, Lot, Corrèze and Gironde departments). In French walnut orchards, the two main historical diseases were bacterial wilt (caused by *Xanthomonas campestris* pv. *juglandis*, walnut blight) causing yield losses of up to 50%. and anthracnose caused by *Ophiognomonia leptostyla* (formerly *Gnomonia juglandis*, Ascomycota, Sordariomycetes). Since 2007, a new fungal disease associated to the *Colletotrichum* genus has appeared in French walnut trees causing fruits browning (anthracnose symptoms) which then become unmarketable^5^.

*Colletotrichum* is a globally distributed plant-associated fungal genus able to cause disease on a wide variety of woody and herbaceous plants^6^, including walnut, on which the pathogen causes a new form of walnut anthracnose. *Colletotrichum acutatum* species complex is a diverse yet relatively closely related group of plant pathogenic fungi within the genus, recently suggested as a model system to study evolution and host specialization in plant pathogens^7^. In 2005, Sreenivasaprasad and Talhinhas reported *C*. *acutatum sensu lato* associated with *J*. *regia*^8^, however no information about the geographic origin and the pathogenicity were reported. The same year Juhasova *et al*. reported the presence of *C*. *gloeosporioides* on walnut fruits in Slovakia, but the importance of the disease was not indicated^9^. Later Damm *et al*. described two *C*. *godetiae* strains associated with walnut: one isolated in Austria and another one of unknown origin^10^. The walnut anthracnose disease caused by *Colletotrichum* spp. is not only restricted to Europe. Recently, 3 reports described *C*. *gloeosporioides sensu lato* as the causal agent of anthracnose on *J*. *regia* in Shandong province, China^11–13^. Zhu *et al*. 2015 also reported leaf spot disease caused by *C*. *fioriniae* on walnut trees in Hechi, Guangxi region, China, which led to severe reductions in nut production^14^. Symptoms are described as water-soaked circular to semi-circular leaf spots, later becoming tan bordered, greyish-white in the centre and dark brown to the margins; lesions are 3 to 4 mm in diameter. Morphological and molecular characterization confirmed the presence of *C*. *fioriniae*. Artificial inoculations and re-isolation of the pathogen from the leaves demonstrated that the causal agent of the disease was *C*. *fioriniae*. Efforts to contain the pathogen spread were made. To date, chemical control has been the main approach to control the disease, although it may lead to environmental concerns and drug resistance in the pathogen^15^. Therefore, identification of resistant cultivars is required.

**Figure 1:**
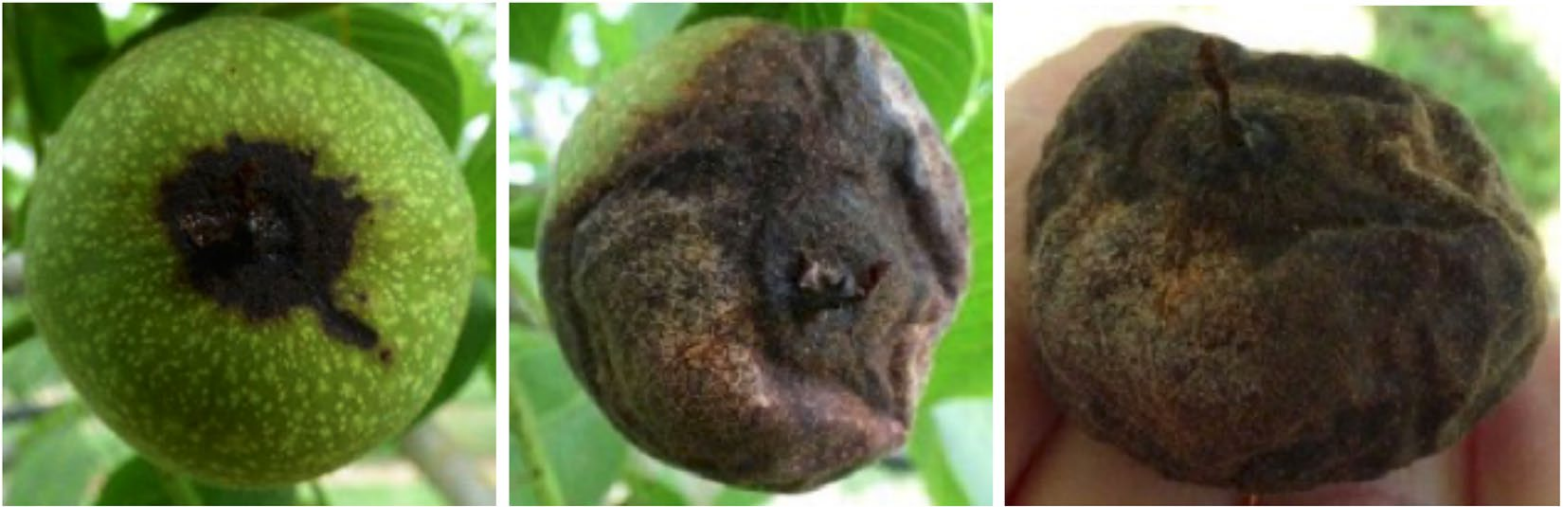
Development of anthracnose symptoms on a walnut fruit. Left: in June small brown to black necrosis, here taking also the aspect of a run-out, appear on young fruit. Centre: around August orange conidial masses can usually be observed. The necrosis has a dry aspect and deforms the husk. Right: The nut can become completely necrotic and deformed, with conidial masses, and falls of the tree.

In France, *Colletotrichum* sp. on walnut has been detected for the first time in 2007 as part of a study regarding the bacteriosis of walnut^5^. Later, in 2011, symptoms of anthracnose appeared on walnut leading to 50–70% losses in nut production; the causal agent was identified as belonging to the *Colletotrichum* genus^5^. To our knowledge, this is the first report of an epidemic event of walnut anthracnose caused by *Colletotrichum* spp. in Europe. The disease mainly affects the surface of the fruit in June and is characterized by small brown or black dry spots. These spots tend to become circular and dark in colour. Orange conidial masses can appear (*i*.*e*., acervuli) on the necrotic spots during the season (depending on meteorological conditions). Eventually, the nut becomes completely necrotic and falls prematurely (Fig. 1).

These symptoms sometimes may be misleading: in the early stages of the disease, necrotic areas can be confused with those caused by *Xanthomonas campestris* pv. *juglandis*; symptoms may also be confused with those caused by *Ophiognomonia leptostyla*, although the spots caused by *O*. *leptostyla* present a typical light-green colouration in the centre^5^.

Considering the severity of the disease on walnut, the focus of the present study was to assess the extent of the genetic and pathogenic diversity of *Colletotrichum* spp. populations associated with walnut anthracnose in France. We used two different approaches: 1. Metabarcoding analysis of *Colletotrichum* spp. diversity in plant tissues; 2. Multi-locus phylogenetic analysis of a collection of *Colletotrichum* spp. isolates established through the work. We selected the most disease-affected area as our sampling zone. Pathogenicity was confirmed by inoculation tests on walnut (cultivar Lara) grown in France.

## Results

### Metabarcoding data

**Figure 2:**
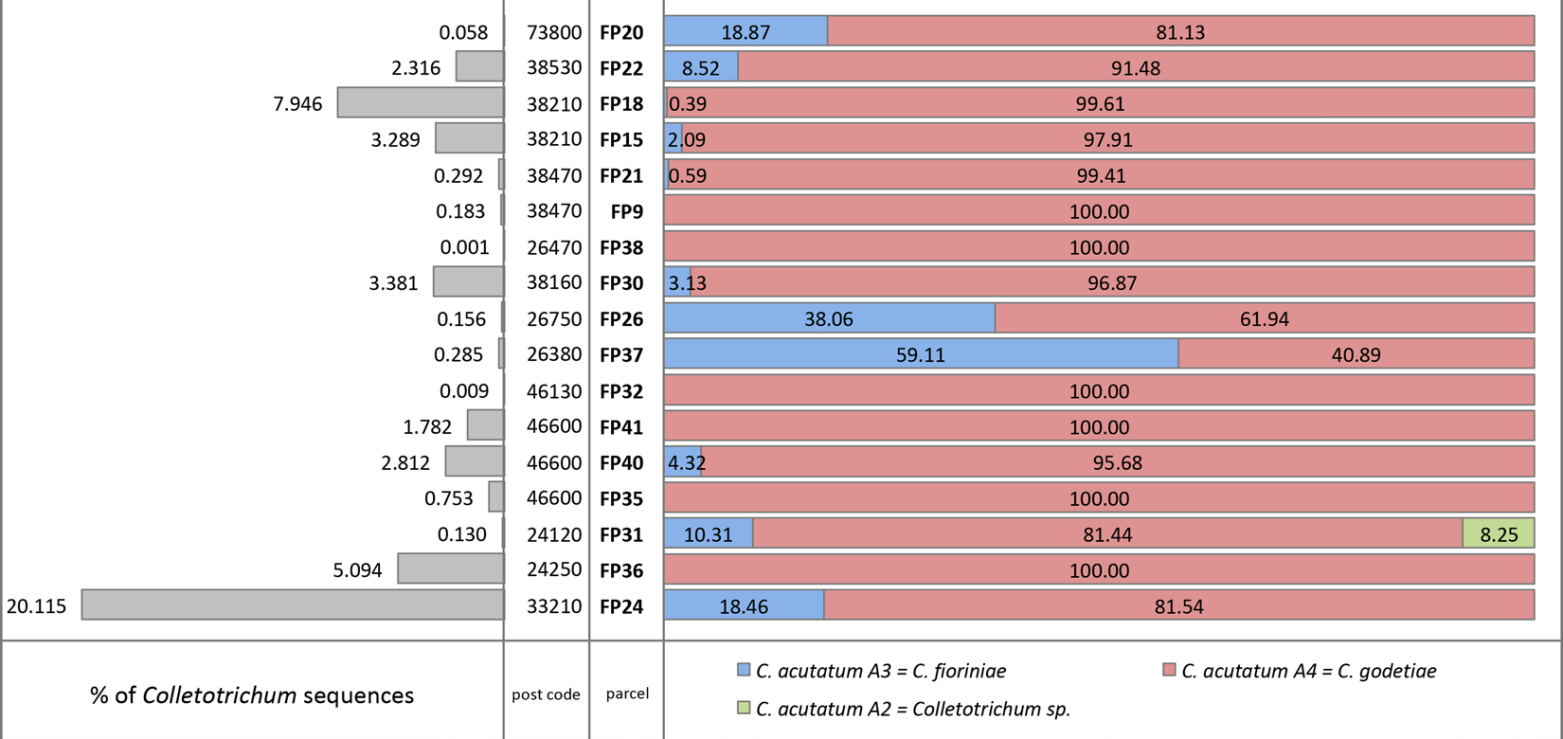
Percentage of occurrence of *Colletotrichum* spp. sequences in the overall number of ITS sequences obtained by metabarcoding (grey bars on the left) and relative percent abundances of *Colletotrichum acutatum sensu lato* ITS groups described by Sreenivasaprasad and Talhinhas^8^, (red, blue and green bars on the right). Post codes and parcel codes are reported in the centre of the figure. Samples are ordered according to geographical position from east to west.

A total of 1,993,250 ITS sequences (from 53,197 to 190,494 per sample) were obtained for the 17 samples collected. A total of 52,663 (2.64%) ITS sequences for the genus *Colletotrichum* were obtained. The overall percentage of *Colletotrichum* species varied from 0.001 in the sample collected in parcel FP38 to 20.12 for sample collected in parcel FP24 (Fig. 2). Only 3 samples had a proportion of *Colletotrichum* ITS sequences greater than 5% (FP24, FP18 and FP36), while 9 samples had abundances below 1% (FP20, FP21, FP9 FP38, FP26, FP37, FP32, FP35 and FP31). Among all the *Colletotrichum* sequences, 3 *C*. *acutatum sensu lato* ITS genetic groups^8^ were detected by metabarcoding approach. *C*. *acutatum sensu lato* was present in all the samples analysed. *C*. *acutatum* group A4, corresponding to *C*. *godetiae*^10^, was present in each sample, with abundances between 61.94 and 100% of the total *Colletotrichum* sequences obtained. Results shown *C*. *godetiae* to be the most abundant species in all samples except FP37, which has *C*. *acutatum* group A3, corresponding to *C*. *fioriniae*^10^, as the most abundant *Colletotrichum* species (40.89% and 59.11% respectively). *C*. *fioriniae* was the second most abundant species found, which is present on 11/17 samples, with abundances between 0.39 and 59.11%. In 5 samples the proportion of *C*. *fioriniae* was above 10%, and in 2 samples was below 1%. A third genetic group belonging to the *C*. *acutatum* species complex, and identified as group A2^8^, was detected. *C*. *acutatum* group A2 was present only in one sample analysed (FP31), representing an 8.25% of all the *Colletotrichum* sequences. Due to the low resolution of the ITS locus in the *C*. *acutatum* species complex and the presence of multiple species in the same genetic group, a correct identification at species level was not possible for this set of sequences.

### Isolate collection

**Figure 3:**
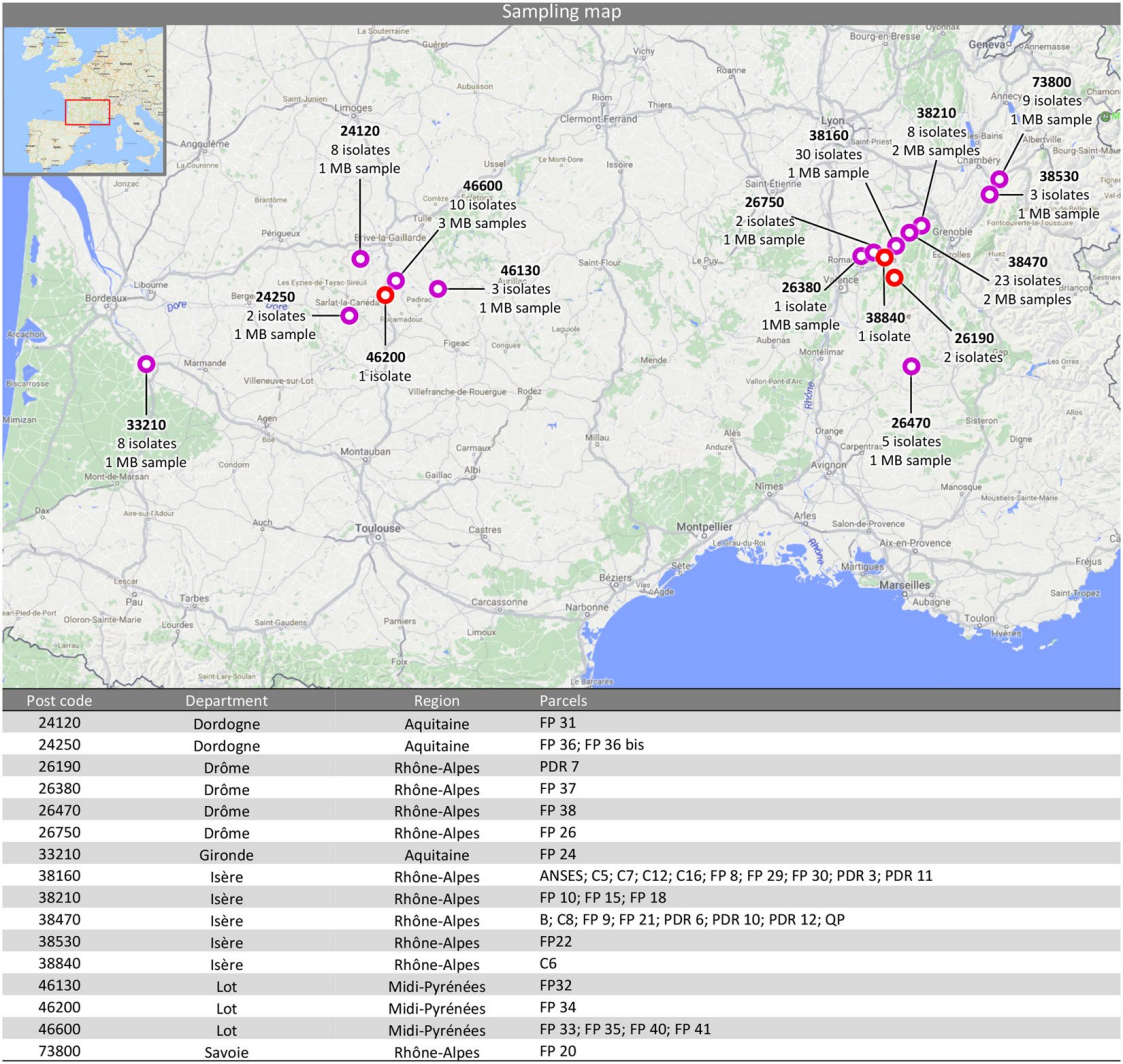
Geographic distribution, postcode and number of samples used to characterize *Colletotrichum* species associated with walnut anthracnose in France. MB corresponding to the metabarcoding samples analysed. Red circles correspond to sites where only classic fungal isolations have been carried out while purple circles correspond to sites where classic isolation and metabarcoding sample have been collected. Geographical information about parcels sampled are reported in the table.

In the present study, a total of 116 samples were obtained (Table 1). Isolate 2015-4-1 was obtained from a scale insect belonging to the Coccoidea superfamily (order Hemiptera), while the other isolates were collected from fruits, buds, leaves and stems of five cultivars and several hybrids of walnut. Eighty-four strains (~72%) were isolated in the South-Eastern (SE) region, while 32 strains (~28%) were isolated in the South-Western (SW) region (Fig. 3).

**Table 1:** *Colletotrichum* spp. strains used in this study with isolation details and GenBank accessions.

Isolate/Culture collection N°	Tissue	Cultivar	Geographic origin	Parcel	ITS	ACT	CHS-1	GAPDH	HIS3	TUB2	GS	CAL	ApMat
*C*. *fioriniae*													
2015-63-1 UBOCC-A-117288	Nut	Franquette	38840, St Bonnet Chavagne	C6	MG589788	MG665997	MG666345	MG666113	MG666461	MG666229			
2015-69-1 UBOCC-A-117423	Nut	Fernor	38160, St Verand	C12	MG589802	MG666011	MG666359	MG666127	MG666475	MG666243			
2015-57-3 UBOCC-A-117425	Nut	Franquette	38470, Cognin les Gorges	PDR 12	MG589804	MG666013	MG666361	MG666129	MG666477	MG666245			
2015-57-1 UBOCC-A-117430	Nut	Franquette	38470, Cognin les Gorges	PDR 12	MG589809	MG666018	MG666366	MG666134	MG666482	MG666250			
2015-52-2 UBOCC-A-117436	Nut	Franquette	26190, St Thomas en Royans	PDR 7	MG589815	MG666024	MG666372	MG666140	MG666488	MG666256			
2015-7-1 UBOCC-A-117437	Nut	Franquette	38160, Chatte	ANSES	MG589817	MG666026	MG666374	MG666142	MG666490	MG666258			
2015-19-2^§^ UBOCC-A-117279	Nut	Parisienne	38210, Cras	FP 15	MG589823	MG666032	MG666380	MG666148	MG666496	MG666264			
2015-24-1 UBOCC-A-117443	Nut	Franquette	73800, Laissaud	FP 20	MG589825	MG666034	MG666382	MG666150	MG666498	MG666266			
2015-24-2 UBOCC-A-117444	Nut	Franquette	73800, Laissaud	FP 20	MG589826	MG666035	MG666383	MG666151	MG666499	MG666267			
2015-25-1 UBOCC-A-117446	Nut	Franquette	38470, Chantesse	FP 21	MG589829	MG666038	MG666386	MG666154	MG666502	MG666270			
2015-26-1^§^ UBOCC-A-117281	Nut	Fernor	38530, La Buissière	FP 22	MG589830	MG666039	MG666387	MG666155	MG666503	MG666271			
2015-28-1 UBOCC-A-117447	Nut	hybrid	33210, Toulenne	FP 24	MG589831	MG666040	MG666388	MG666156	MG666504	MG666272			
2015-34-4 UBOCC-A-117452	Nut	Franquette	38160, St Romans	FP 30	MG589837	MG666046	MG666394	MG666162	MG666510	MG666278			
2015-41-1^§^ UBOCC-A-117284	Nut	Franquette	24250, St Cybranet	FP 36 bis	MG589843	MG666052	MG666400	MG666168	MG666516	MG666284			
2015-41-2 UBOCC-A-117457	Nut	Franquette	24250, St Cybranet	FP 36 bis	MG589844	MG666053	MG666401	MG666169	MG666517	MG666285			
2015-43-2 UBOCC-A-117459	Nut	Lara	26470, La Motte Chalancon	FP 38	MG589847	MG666056	MG666404	MG666172	MG666520	MG666288			
2015-43-3 UBOCC-A-117460	Nut	Lara	26470, La Motte Chalancon	FP 38	MG589848	MG666057	MG666405	MG666173	MG666521	MG666289			
2015-43-4 UBOCC-A-117461	Nut	Lara	26470, La Motte Chalancon	FP 38	MG589849	MG666058	MG666406	MG666174	MG666522	MG666290			
2016-3-1	Bud	Franquette	73800, Laissaud	FP 20	MG589858	MG666067	MG666415	MG666183	MG666530	MG666299			
2016-3-2	Bud	Franquette	73800, Laissaud	FP 20	MG589859	MG666068	MG666416	MG666184	MG666531	MG666300			
2016-3-3	Bud	Franquette	73800, Laissaud	FP 20	MG589860	MG666069	MG666417	MG666185	MG666532	MG666301			
2016-4-2	Bud	hybrid	33210, Toulenne	FP 24	MG589864	MG666073	MG666421	MG666189	MG666536	MG666305			
2016-4-3	Bud	hybrid	33210, Toulenne	FP 24	MG589865	MG666074	MG666422	MG666190	MG666537	MG666306			
2016-6-1	Nut	hybrid	33210, Toulenne	FP 24	MG589870	MG666079	MG666427	MG666195	MG666542	MG666311			
2016-11-2	Bud	hybrid	33210, Toulenne	FP 24	MG589878	MG666087	MG666435	MG666203	MG666550	MG666319			
2016-12-1	Bud	hybrid	26750, Geyssans	FP 26	MG589879	MG666088	MG666436	MG666204	MG666551	MG666320			
2016-13-3	Bud	Franquette	24120, Terrasson La Villedieu	FP 31	MG589882	MG666091	MG666439	MG666207	MG666554	MG666323			
2016-13-4	Bud	Franquette	24120, Terrasson La Villedieu	FP 31	MG589883	MG666092	MG666440	MG666208	MG666555	MG666324			
2016-14-1	Bud	Fernor	46600, Montvalent	FP 35	MG589884	MG666093	MG666441	MG666209	MG666556	MG666325			
2016-14-3	Bud	Fernor	46600, Montvalent	FP 35	MG589886	MG666095	MG666443	MG666211	MG666558	MG666327			
2016-14-4	Bud	Fernor	46600, Montvalent	FP 35	MG589887	MG666096	MG666444	MG666212	MG666559	MG666328			
2016-16-1	Bud	Parisienne	38210, Cras	FP 15	MG589889	MG666098	MG666446	MG666214	MG666561	MG666330			
2016-21-3	Bud	Fernor	46130, Puybrun	FP 32	MG589899	MG666108	MG666456	MG666224	MG666571	MG666340			
2016-23-1	Bud	Lara	26470, La Motte Chalancon	FP 38	MG589900	MG666109	MG666457	MG666225	MG666572	MG666341			
*C*. *godetiae*													
2015-62-1 UBOCC-A-117411	Nut	Franquette	38160, Chatte	C5	MG589789	MG665998	MG666346	MG666114	MG666462	MG666230			
2015-73-1 UBOCC-A-117412	Nut	Franquette	38160, Chatte	C16	MG589790	MG665999	MG666347	MG666115	MG666463	MG666231			
2015-73-5 UBOCC-A-117413	Nut	Franquette	38160, Chatte	C16	MG589791	MG666000	MG666348	MG666116	MG666464	MG666232			
2015-73-4 UBOCC-A-117289	Nut	Franquette	38160, Chatte	C16	MG589792	MG666001	MG666349	MG666117	MG666465	MG666233			
2015-64-1 UBOCC-A-117414	Nut	Franquette	38160, Chatte	C7	MG589793	MG666002	MG666350	MG666118	MG666466	MG666234			
2015-65-1 UBOCC-A-117415	Leaf	Franquette	38470, L’Albenc	C8	MG589794	MG666003	MG666351	MG666119	MG666467	MG666235			
2015-51-1 UBOCC-A-117416	Nut	Franquette	38470, Beaulieu	PDR 6	MG589795	MG666004	MG666352	MG666120	MG666468	MG666236			
2015-48-2 UBOCC-A-117417	Nut	Franquette	38160, Chevrières	PDR 3	MG589796	MG666005	MG666353	MG666121	MG666469	MG666237			
2015-48-1 UBOCC-A-117418	Nut	Franquette	38160, Chevrières	PDR 3	MG589797	MG666006	MG666354	MG666122	MG666470	MG666238			
2015-48-10 UBOCC-A-117419	Nut	Franquette	38160, Chevrières	PDR 3	MG589798	MG666007	MG666355	MG666123	MG666471	MG666239			
2015-48-9 UBOCC-A-117420	Nut	Franquette	38160, Chevrières	PDR 3	MG589799	MG666008	MG666356	MG666124	MG666472	MG666240			
2015-48-8 UBOCC-A-117421	Nut	Franquette	38160, Chevrières	PDR 3	MG589800	MG666009	MG666357	MG666125	MG666473	MG666241			
2015-48-7 UBOCC-A-117422	Nut	Franquette	38160, Chevrières	PDR 3	MG589801	MG666010	MG666358	MG666126	MG666474	MG666242			
2015-73-6 UBOCC-A-117424	Nut	Franquette	38160, Chatte	C16	MG589803	MG666012	MG666360	MG666128	MG666476	MG666244			
2015-48-11 UBOCC-A-117426	Nut	Franquette	38160, Chevrières	PDR 3	MG589805	MG666014	MG666362	MG666130	MG666478	MG666246			
2015-73-3 UBOCC-A-117427	Nut	Franquette	38160, Chatte	C16	MG589806	MG666015	MG666363	MG666131	MG666479	MG666247			
2015-48-5 UBOCC-A-117428	Nut	Franquette	38160, Chevrières	PDR 3	MG589807	MG666016	MG666364	MG666132	MG666480	MG666248			
2015-57-2 UBOCC-A-117429	Nut	Franquette	38470, Cognin les Gorges	PDR 12	MG589808	MG666017	MG666365	MG666133	MG666481	MG666249			
2015-48-3 UBOCC-A-117431	Nut	Franquette	38160, Chevrières	PDR 3	MG589810	MG666019	MG666367	MG666135	MG666483	MG666251			
2015-48-4 UBOCC-A-117432	Nut	Franquette	38160, Chevrières	PDR 3	MG589811	MG666020	MG666368	MG666136	MG666484	MG666252			
2015-56-1 UBOCC-A-117433	Nut	Franquette	38160, St Appolinard	PDR 11	MG589812	MG666021	MG666369	MG666137	MG666485	MG666253			
2015-55-1 UBOCC-A-117434	Nut	Franquette	38470, Chantesse	PDR 10	MG589813	MG666022	MG666370	MG666138	MG666486	MG666254			
2015-52-1 UBOCC-A-117435	Nut	Franquette	26190, St Thomas en Royans	PDR 7	MG589814	MG666023	MG666371	MG666139	MG666487	MG666255			
2015-4-1 UBOCC-A-117277	Insect	insect	38160, Chatte	-	MG589816	MG666025	MG666373	MG666141	MG666489	MG666257			
2015-10-1 UBOCC-A-117438	Nut	Franquette	38160, St Appolinard	FP 8	MG589818	MG666027	MG666375	MG666143	MG666491	MG666259			
2015-11-1 UBOCC-A-117439	Nut	Franquette	38470, Beaulieu	FP 9	MG589819	MG666028	MG666376	MG666144	MG666492	MG666260			
2015-11-2 UBOCC-A-117440	Nut	Franquette	38470, Beaulieu	FP 9	MG589820	MG666029	MG666377	MG666145	MG666493	MG666261			
2015-12-1 UBOCC-A-117441	Nut	Parisienne	38210, Tullins	FP 10	MG589821	MG666030	MG666378	MG666146	MG666494	MG666262			
2015-19-1^§^ UBOCC-A-117278	Nut	Parisienne	38210, Cras	FP 15	MG589822	MG666031	MG666379	MG666147	MG666495	MG666263			
2015-22-1 UBOCC-A-117442	Nut	Franquette	38210, Cras	FP 18	MG589824	MG666033	MG666381	MG666149	MG666497	MG666265			
2015-24-3^§^ UBOCC-A-117280	Nut	Franquette	73800, Laissaud	FP 20	MG589827	MG666036	MG666384	MG666152	MG666500	MG666268			
2015-24-4 UBOCC-A-117445	Nut	Franquette	73800, Laissaud	FP 20	MG589828	MG666037	MG666385	MG666153	MG666501	MG666269			
2015-30-1 UBOCC-A-117282	Nut	Fernor	26750, Geyssans	FP 26	MG589832	MG666041	MG666389	MG666157	MG666505	MG666273			
2015-33-1 UBOCC-A-117448	Nut	Chandler	38160, Chatte	FP 29	MG589833	MG666042	MG666390	MG666158	MG666506	MG666274			
2015-34-1 UBOCC-A-117449	Nut	Franquette	38160, St Romans	FP 30	MG589834	MG666043	MG666391	MG666159	MG666507	MG666275			
2015-34-2 UBOCC-A-117450	Nut	Franquette	38160, St Romans	FP 30	MG589835	MG666044	MG666392	MG666160	MG666508	MG666276			
2015-34-3 UBOCC-A-117451	Nut	Franquette	38160, St Romans	FP 30	MG589836	MG666045	MG666393	MG666161	MG666509	MG666277			
2015-35-1 UBOCC-A-117453	Nut	Franquette	24120, Terrasson La Villedieu	FP 31	MG589838	MG666047	MG666395	MG666163	MG666511	MG666279			
2015-35-2 UBOCC-A-117283	Nut	Franquette	24120, Terrasson La Villedieu	FP 31	MG589839	MG666048	MG666396	MG666164	MG666512	MG666280			
2015-37-1 UBOCC-A-117454	Nut	Lara	46600, St Denis lès Martel	FP 33	MG589840	MG666049	MG666397	MG666165	MG666513	MG666281			
2015-38-1 UBOCC-A-117455	Nut	Franquette	46200, Pinsac	FP 34	MG589841	MG666050	MG666398	MG666166	MG666514	MG666282			
2015-39-1 UBOCC-A-117456	Nut	Fernor	46600, Montvalent	FP 35	MG589842	MG666051	MG666399	MG666167	MG666515	MG666283			
2015-39-2^§^ UBOCC-A-117285	Nut	Fernor	46600, Montvalent	FP 35	MG589845	MG666054	MG666402	MG666170	MG666518	MG666286			
2015-43-1 UBOCC-A-117458	Nut	Lara	26470, La Motte Chalancon	FP 38	MG589846	MG666055	MG666403	MG666171	MG666519	MG666287			
2016-1-1	Bud	Franquette	38470, Chantesse	B	MG589850	MG666059	MG666407	MG666175	MG666523	MG666291			
2016-1-2	Bud	Franquette	38470, Chantesse	B	MG589851	MG666060	MG666408	MG666176	MG666524	MG666292			
2016-1-5	Bud	Franquette	38470, Chantesse	B	MG589853	MG666062	MG666410	MG666178	MG666525	MG666294			
2016-2-1	Bud	Franquette	38470, L’Albenc	QP	MG589854	MG666063	MG666411	MG666179	MG666526	MG666295			
2016-2-2	Bud	Franquette	38470, L’Albenc	QP	MG589855	MG666064	MG666412	MG666180	MG666527	MG666296			
2016-2-3	Bud	Franquette	38470, L’Albenc	QP	MG589856	MG666065	MG666413	MG666181	MG666528	MG666297			
2016-2-4	Bud	Franquette	38470, L’Albenc	QP	MG589857	MG666066	MG666414	MG666182	MG666529	MG666298			
2016-3-4	Bud	Franquette	73800, Laissaud	FP 20	MG589861	MG666070	MG666418	MG666186	MG666533	MG666302			
2016-3-5	Bud	Franquette	73800, Laissaud	FP 20	MG589862	MG666071	MG666419	MG666187	MG666534	MG666303			
2016-4-1	Bud	hybrid	33210, Toulenne	FP 24	MG589863	MG666072	MG666420	MG666188	MG666535	MG666304			
2016-4-4	Bud	hybrid	33210, Toulenne	FP 24	MG589866	MG666075	MG666423	MG666191	MG666538	MG666307			
2016-5-2	Bud	Fernor	46600, Montvalent	FP 35	MG589868	MG666077	MG666425	MG666193	MG666540	MG666309			
2016-5-3	Bud	Fernor	46600, Montvalent	FP 35	MG589869	MG666078	MG666426	MG666194	MG666541	MG666310			
2016-7-1	Stem	Franquette	38160, Chatte	ANSES	MG589871	MG666080	MG666428	MG666196	MG666543	MG666312			
2016-8-1	Bud	Franquette	38210, Cras	FP 18	MG589872	MG666081	MG666429	MG666197	MG666544	MG666313			
2016-9-1	Bud	Franquette	38470, Chantesse	FP 21	MG589873	MG666082	MG666430	MG666198	MG666545	MG666314			
2016-9-2	Bud	Franquette	38470, Chantesse	FP 21	MG589874	MG666083	MG666431	MG666199	MG666546	MG666315			
2016-10-1	Bud	Fernor	38530, La Buissière	FP 22	MG589875	MG666084	MG666432	MG666200	MG666547	MG666316			
2016-10-2	Bud	Fernor	38530, La Buissière	FP 22	MG589876	MG666085	MG666433	MG666201	MG666548	MG666317			
2016-11-1	Bud	hybrid	33210, Toulenne	FP 24	MG589877	MG666086	MG666434	MG666202	MG666549	MG666318			
2016-13-1	Bud	Franquette	24120, Terrasson La Villedieu	FP 31	MG589880	MG666089	MG666437	MG666205	MG666552	MG666321			
2016-13-2	Bud	Franquette	24120, Terrasson La Villedieu	FP 31	MG589881	MG666090	MG666438	MG666206	MG666553	MG666322			
2016-14-2	Bud	Fernor	46600, Montvalent	FP 35	MG589885	MG666094	MG666442	MG666210	MG666557	MG666326			
2016-15-1	Bud	Franquette	26380, Peyrins	FP 37	MG589888	MG666097	MG666445	MG666213	MG666560	MG666329			
2016-16-2	Bud	Parisienne	38210, Cras	FP 15	MG589890	MG666099	MG666447	MG666215	MG666562	MG666331			
2016-17-1	Bud	Franquette	38210, Cras	FP 18	MG589891	MG666100	MG666448	MG666216	MG666563	MG666332			
2016-18-1	Bud	Franquette	38470, Chantesse	FP 21	MG589892	MG666101	MG666449	MG666217	MG666564	MG666333			
2016-19-1	Bud	Franquette	38160, St Romans	FP 30	MG589893	MG666102	MG666450	MG666218	MG666565	MG666334			
2016-19-2	Bud	Franquette	38160, St Romans	FP 30	MG589894	MG666103	MG666451	MG666219	MG666566	MG666335			
2016-20-1	Bud	Franquette	24120, Terrasson La Villedieu	FP 31	MG589895	MG666104	MG666452	MG666220	MG666567	MG666336			
2016-20-2	Bud	Franquette	24120, Terrasson La Villedieu	FP 31	MG589896	MG666105	MG666453	MG666221	MG666568	MG666337			
2016-21-1	Bud	Fernor	46130, Puybrun	FP 32	MG589897	MG666106	MG666454	MG666222	MG666569	MG666338			
2016-21-2	Bud	Fernor	46130, Puybrun	FP 32	MG589898	MG666107	MG666455	MG666223	MG666570	MG666339			
2016-24-1	Bud	Franquette	38470, Beaulieu	FP 9	MG589901	MG666110	MG666458	MG666226	MG666573	MG666342			
2016-24-2	Bud	Franquette	38470, Beaulieu	FP 9	MG589902	MG666111	MG666459	MG666227	MG666574	MG666343			
2016-24-3	Bud	Franquette	38470, Beaulieu	FP 9	MG589903	MG666112	MG666460	MG666228	MG666575	MG666344			
*C*. *nymphaeae*													
2016-5-1^§^ UBOCC-A-117287	Bud	Fernor	46600, Montvalent	FP 35	MG589867	MG666076	MG666424	MG666192	MG666539	MG666308	—	—	—
*Colletotrichum gloeosporioides sensu lato*													
2016-1-3^§^ UBOCC-A-117286	Bud	Franquette	38470, Chantesse	B	MG589852	MG666061	MG666409	MG666177	—	MG666293	MG666577	MG666576	MG666578

^§^Strains used for pathogenicity tests.

On PDA plates incubated at room temperature (~20 °C), cultures have two main morphological types.

**Figure 4:**
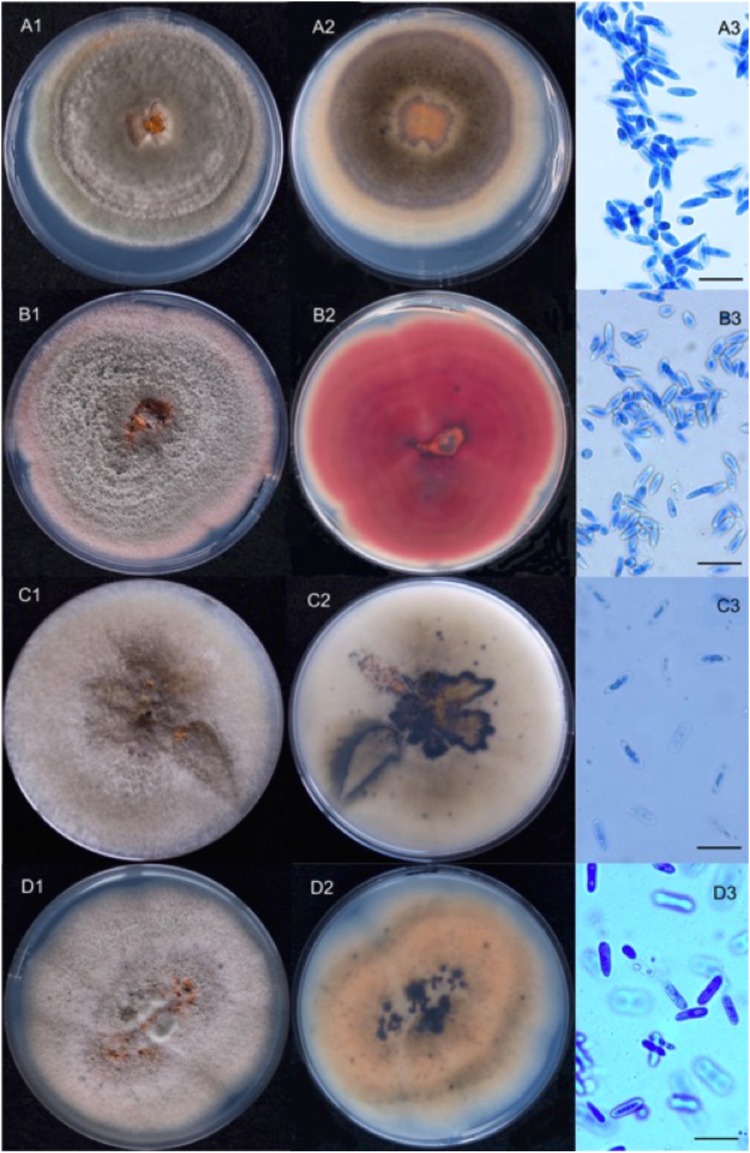
Ten-days *Colletotrichum* spp. cultures grown on PDA and isolated from nuts lesions. 1: upper side, 2: reverse, 3: conidia of A: *C*. *godetiae* (2015-24-3); B: *C*. *fioriniae* (2015-41-1); C: *C*. *gloeosporioides sensu lato* (2016-1-3); D: *C*. *nymphaeae* (2016-5-1). Conidia have been stained by cotton blue (scale bar: 20 µm).

The first morphotype was light grey, with cottony aerial mycelium becoming darker with age and with reverse colours ranging from brownish orange to dark grey with black spots (Fig. 4A1,A2). The majority of isolates with this morphology were later characterized as *C*. *godetiae*. The second morphotype was white to light grey on the upper side and brownish pink to vinaceous with black spots on reverse (Fig. 4B1,B2). All isolates with this morphology were later characterized as *C*. *fioriniae*. In our study two other species were isolated from walnuts, one isolate (2016-1-3) belongs to *C*. *gloeosporioides* species complex, and one isolate (2016-5-1) was identified as *C*. *nymphaeae*; the morphotypes of these two isolates are quite similar to those of the first type, but the isolate 2016-5-1 has a more orange reverse (Fig. 4C1,C2,D1,D2). When cultivated under daylight conditions the colonies showed diurnal zonation sometimes visible on the reverse side as concentric dark circles (Fig. 4A2,B2). Whatever their morphology, all the cultures have dark melanised structures similar to acervuli that oozed orange-coloured conidia. Conidia were hyaline and unicellular, cylindrical to fusiform, pointed at one or both ends (except for those from isolate 2016-5-1 which show both ends rounded), and measured 10.0 to 14.0 μm × 3.0 to 4 μm (Fig. 4A3,B3,C3 and D3) (at least 20 conidia were measured for each isolate). Both cultural and morphological characteristics were similar to those described for *C*. *acutatum sensu lato*^8^ with the exception of isolate 2016-5-1, for which conidial morphology is similar to that of *C*. *gloeosporioides sensu lato*^16^.

### Species identification and genetic diversity

In order to identify the species complex of each isolate obtained during this study, a phylogenetic tree of the *Colletotrichum* genus was built. The multi-locus analysis using the ITS, GAPDH and TUB2 performed on the 116 isolates of *Colletotrichum* spp. associated with walnut-growing regions revealed that 115 isolates belonged to the *C*. *acutatum* species complex and 1 isolate to the *C*. *gloeosporioides* species complex. For *C*. *acutatum* species, the phylogenetic analysis of 115 isolates and 39 reference isolates, using *C*. *orchidophilum* as outgroup, was performed. The multi-locus sequence alignment obtained concatenating ITS, CHS-1, TUB2, ACT, HIS3 and GAPDH loci, consisted of 2124 characters, of which 1591 were conserved, 303 were parsimony-informative and 208 were singleton (Supplementary Table 1).

**Figure 5:**
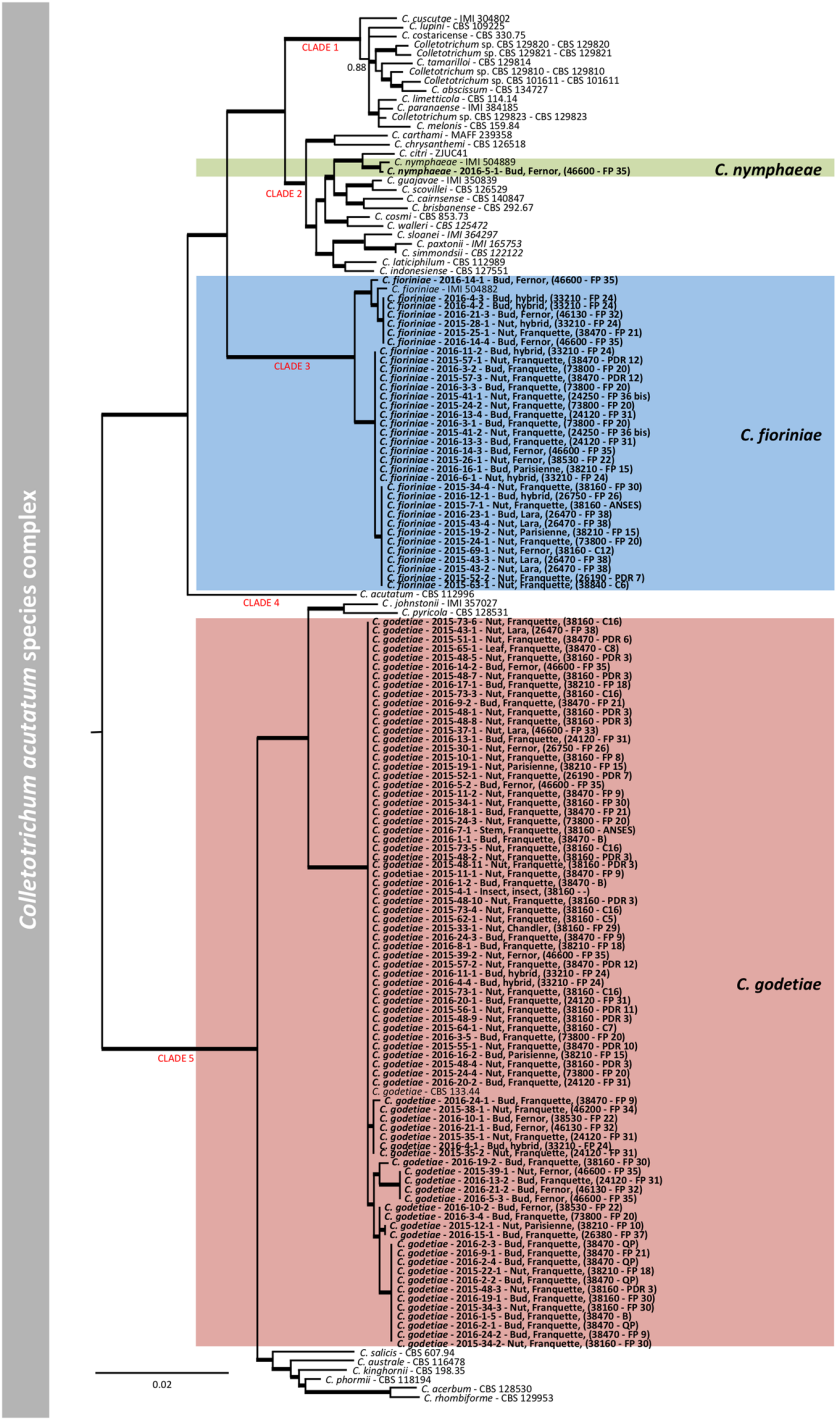
Bayesian inference phylogenetic tree reconstructed from a combined ITS, HIS3, GAPDH, CHS-1, TUB2 and ACT sequence alignment of 154 isolates of the *C*. *acutatum* species complex including the outgroup. Bayesian posterior probability (BPP) values (above 0.50) are shown at the nodes. The thickened nodes represent BPP of 1. Isolates obtained in this study are emphasized in bold font. *C*. *orchidophilum* CBS 632.8 is used as outgroup. Main clades within the *C*. *acutatum* species complex from Damm *et al*. (2012) are indicated in red. The scale bar represents the number of expected substitutions per site. Information such as tissue sampled, cultivar and geographic information (in brackets) for the isolates obtained in this work are reported.

**Figure 6:**
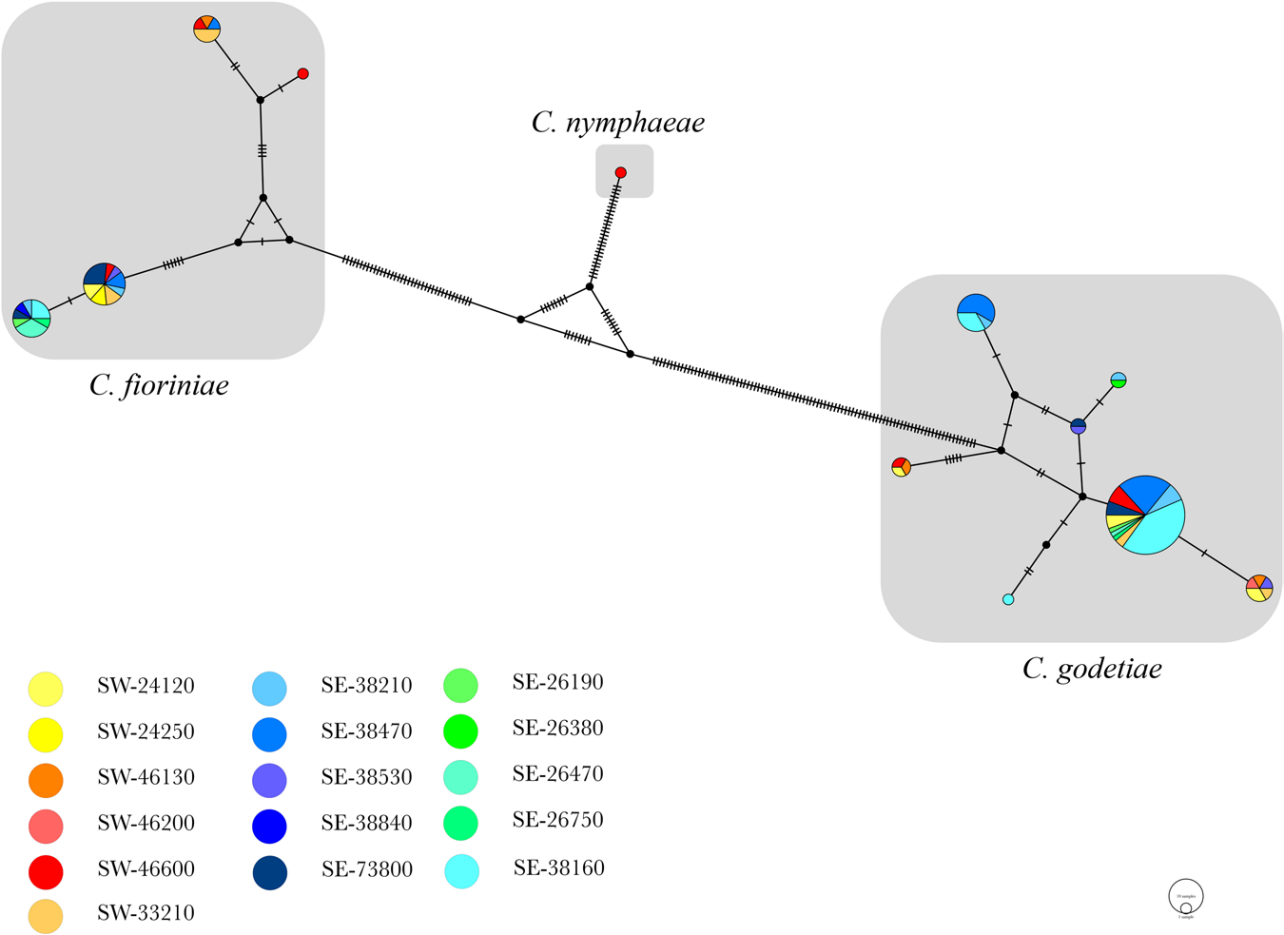
Median-joining network of 12 *Colletotrichum acutatum* species haplotypes based on concatenation of ITS, HIS3, GAPDH, CHS-1, TUB2 and ACT sequences alignments. Circles areas are proportional to the number of strains with a specific haplotype. Segments reported in the connecting lines represent number of mutations between haplotypes. Circles slices area is proportional to the number of strains isolates from a specific geographic area whereas colours indicate the geographic origin according to legend (from yellow to red indicate south west (SW) of France while from green to blue indicate south east (SE) of France).

Based on the multi-locus phylogenetic analysis (Fig. 5), the 115 *C*. *acutatum sensu lato* isolates belong to three different species: *C*. *godetiae* (*C*. *acutatum* group A4), *C*. *fioriniae* (*C*. *acutatum* group A3) and *C*. *nymphaeae* (*C*. *acutatum* group A2). *C*. *godetiae*, with 80 isolates (69% of the samples), was the most abundant species, including the isolate 2015-4-1, isolated from an insect in 38160. Considering all the isolates, *C*. *godetiae* was identified in 14 out of 16 geographical sites with 100% isolates of *C*. *godetiae* identified in 26380 (SE) and 46200 (SW). *C*. *fioriniae* was the second most abundant species with 34 isolates (29.3% of the samples) found in 14 out of 16 sites, among which 24250 (SW) and 28840 (SE) resulted in 100% samples of *C*. *fioriniae*. Finally, one isolate (2016-5-1), which resulted from 46600 (SW), was identified as *C*. *nymphaeae* (Fig. 5). Except for the sites where *C*. *godetiae* was not present, and excluding the ones with 100% abundance, the presence of *C*. *godetiae* in the sites varied from 20% (26470, SE) to 90% (38160, SE), while the abundance of *C*. *fioriniae* varied from 10% in 38160 (SE) to 80% in 26470 (SW). Considering the two main regions, *C*. *godetiae* was the most abundant species in both SE and SW areas with 56.25% and 73.81% abundance, respectively. The haplotype network analysis performed over the 115 isolates of *C*. *acutatum sensu lato* resulted in 4 different haplotypes of *C*. *fioriniae*, 7 different haplotypes of *C*. *godetiae* and 1 haplotype of *C*. *nymphaeae* (Fig. 6). Their geographical distribution revealed 7 haplotypes in SW regions, covering all the three species, and 9 haplotypes in SE regions, covering *C*. *fioriniae* and *C*. *godetiae*. Three haplotypes were exclusively present in the SW regions and covered all the three species, while five haplotypes were present in the SE regions only, covering the *C*. *fioriniae* and *C*. *godetiae* species. A total of 17 nucleotide variations were counted in both populations of *C*. *fioriniae* and *C*. *godetiae*. The AMOVA results (Table 2) showed that more than 82% of molecular variation is contained within the populations (isolates from each field), and a significant (P < 0.01) differentiation was detected among the populations relative to the total population (F_ST_ = 0.179) and among populations within groups (F_SC_ = 0.121). Even showing different haplotypes structure (Fig. 6), differentiation was not significant (P = 0.072, F_CT_ = 0.066) among groups (geographical regions), which indicates that these regions must be connected by some mechanism of dispersion.

**Table 2:** Analysis of molecular variance (AMOVA) results showing the variance among groups (Geographical areas: SW and SE) and populations (parcels).

Source of variation	d.f.	Sum of squares	Variance components	Percentage of variation	P	Statistics
Among groups	1	1.956	0.02562	6.59	0.072	F_CT_ = 0.06591
Among populations within groups	14	8.437	0.04459	11.34	<0.01	F_SC_ = 0.12138
Within populations	98	31.633	0.32279	82.07	<0.01	F_ST_ = 0.17929

**Figure 7:**
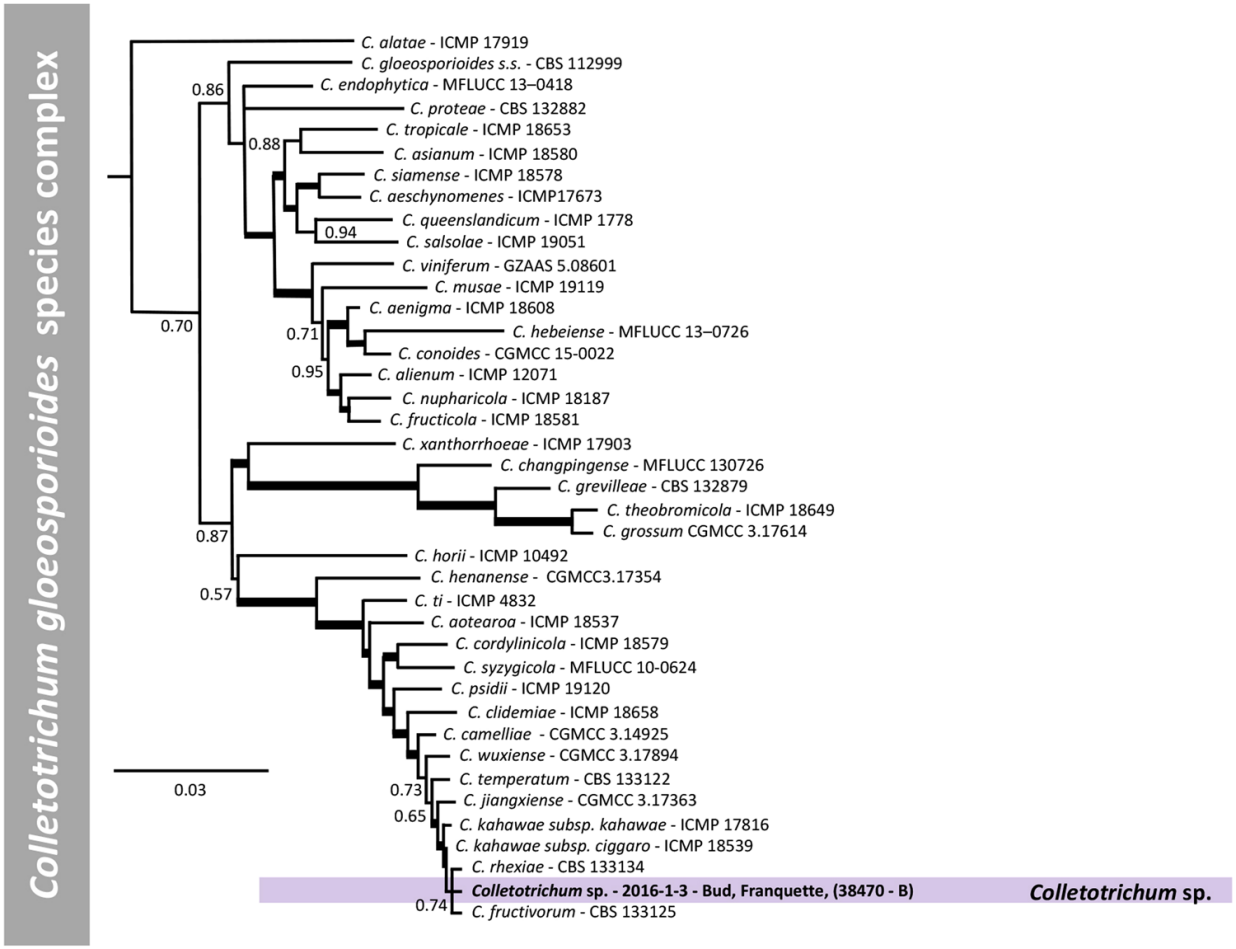
Bayesian inference phylogenetic tree reconstructed from a combined ITS, GAPDH, CHS-1, ACT, TUB2, GS, SOD2, ApMAT and CAL sequence alignment of 40 isolates of the *C*. *gloeosporioides* species complex including the outgroup. Bayesian posterior probability (BPP) values (above 0.50) are shown at the nodes. The thickened nodes represent BPP of 1. Isolates obtained in this study are emphasized in bold font. *Colletotrichum sydowii* CBS 135819 is used as outgroup. The scale bar represents the number of expected substitutions per site. Information such as tissue sampled, cultivar and geographic information (in brackets) for the isolates obtained in this work are reported.

For *C*. *gloeosporioides sensu lato*, 1 isolate and 39 reference isolates, with *C*. *sydowii* as outgroup, were analysed. Phylogenetic analysis was performed on a multi-locus concatenated sequence alignment (ITS, CHS-1, CAL, ACT, SOD2, TUB2, GS, GAPDH and ApMAT locus) resulting in 5716 characters, of which 3658 were conserved, 768 parsimony-informative and 1051 singletons (Supplementary Table 1). Based on the multi-locus phylogenetic analysis, the *C*. *gloeosporioides sensu lato* isolate (2016-1-3) deriving from site 38470, in the SE region, does not belong to any accepted species and is closely related to *C*. *rhexiae* and *C*. *fructivorum* (Fig. 7).

### Pathogenicity tests

Nineteen days after inoculation, all fruits clearly showed necrotic lesions, all strains tested were pathogenic on walnuts fruits; Koch’s postulates, therefore, were verified.

When diameters of necrotic lesions were submitted to ANOVA, all isolates produced lesions whose diameter was significantly bigger than those on control (P = 0.0001).

**Figure 8:**
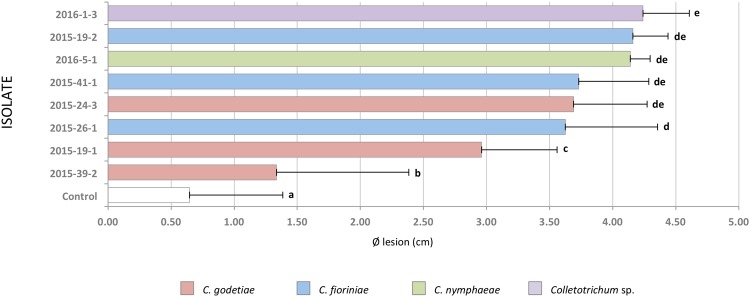
Histograms showing average lesions size of 8 *Colletotrichum* reference isolates on walnut fruits (cultivar Lara). Bars indicate the average diameters of the lesion in cm. Standard deviations are reported as lines at the end of each bar. Letters at the extreme of each bar indicate significant differences based on ANOVA Tukey post hoc test results.

Data were then submitted to *post hoc* Tukey’s test whose results are showed in Fig. 8. Generally, isolates could be divided into two groups: the first including *C*. *fioriniae* 2015-26-1, *C*. *godetiae* 2015-24-3, *C*. *fioriniae* 2015-41-1, *C*. *nymphaeae* 2016-5-1, *C*. *fioriniae* 2015-19-2 and *C*. *gloeosporioides sensu lato* 2016-1-3 that showed no significant intra-grouping differences among them; the second included two *C*. *godetiae* strains (2015-39-2 and 2015-19-1) that caused lesions significantly smaller than those produced by the other isolates but significantly larger than controls.

## Discussion

In 2011, an epidemic of anthracnose on walnut was observed in France. This was shown to be caused by members of the genus *Colletotrichum*^5^, leading to 50–70% of losses with some orchards experiencing 100% losses. In the past decade, anthracnose on walnut caused by *Colletotrichum* spp. was also reported in the Shandong province and in the Guangxi region, in China^12–14^. However, *Colletotrichum* species causing epidemic infections of walnut anthracnose in Europe have never been characterized. Information regarding the presence of *Colletotrichum* spp. on walnut in Europe is scarce; however one strain of *C*. *godetiae* and one of *C*. *gloeosporioides* have been associated with this plant in Austria^10^ and Slovakia^9^ respectively. Hence, there was a need to characterize the species of *Colletotrichum* associated with walnut, which was the basis of the present study. The current study represents the first identification of *Colletotrichum* species associated with anthracnose of walnut in France using a metabarcoding and a multi-locus phylogenetic combined approach.

Molecular identification of the pathogenic species associated with walnut provides a useful tool to help to understand the distribution and the interactions between the host and its pathogens. In this study, a total of 116 isolates were obtained from infected walnuts tissues. In France, walnut is mainly cultivated in the Auvergne-Rhône-Alpes region in SE and in the Occitanie and Nouvelle-Aquitaine regions in SW. Samples were collected where the disease incidence was higher, mainly in the former Rhône-Alpes region for SE samples and between Aquitaine, Midi-Pyrénées and Limousin former regions for SW samples. Moreover, parts of these areas were sampled and used for metabarcoding analysis.

The multi-locus characterization method led to the identification of four different species: 80 isolates of *C*. *godetiae* (69%), 34 isolates of *C*. *fioriniae* (29.3%), 1 isolate of *C*. *nymphaeae* (0.86%) and 1 isolate of *C*. *gloeosporioides sensu lato* (2016-1-3, 0.86%). These results are coherent with data obtained from the metabarcoding analysis where the most abundant sequences belong to *C*. *acutatum* group A4 (*C*. *godetiae*, 17/17 of the samples), corresponding to 89.88% of the total *Colletotrichum* sequences, followed by *C*. *acutatum* group A3 (*C*. *fioriniae*, 11/17 of the samples), corresponding to 9.64% of the *Colletotrichum* sequences obtained.

Metabarcoding analysis is a powerful DNA sequencing technique that provides a realistic approximation of the quantitative presence of species in a sample. It is also a useful tool to characterize the species recovered in a sample^17^.

However, it is important to highlight that metabarcoding analysis, due to the presence of chimeric sequences or differences in template DNA copy number, can suffer from biases which may lead to an overestimation or underestimation of the species present in a sample^17^. Moreover a metabarcoding approach can detect false positive due to the persistence of DNA in the environment after cells have lost viability^18^. On the other hand, fungal isolation methods are suitable to characterize the species of a sample and to cover its variability, since they are based on phenotypic characters that may be highly selective. Therefore, in order to correctly identify the cultivable pathogenic species associated to a specific host, metabarcoding analysis should always be coupled with isolation methods.

Whilst being accepted widely as the universal fungal barcode region, the ITS region is not able to delimit species with the genus *Colletotrichum*, and especially not within its species complexes such as *C*. *acutatum sensu lato*. In contrast, the use of fungal isolation methods coupled with the multilocus genetic characterization enabled the definition of the *C*. *acutatum* A2 genetic group as *C*. *nymphaeae*. Furthermore, fungal isolations allowed the recovery of a fourth *Colletotrichum* species belonging to the *C*. *gloeosporioides* species complex and closely related to *C*. *rhexiae* and *C*. *fructivorum*.

Samples derived from the southern part of France, were mapped and divided on the basis of their geographical origin. The two most representative species, *C*. *godetiae* and *C*. *fioriniae*, do not show a uniform distribution between the two areas, and no significant differentiation was found at the haplotype level between the two areas. All things considered, on the basis of the samples we had and the results we obtained, we could not find any correlation that could indicate a common origin of the haplotypes where the disease initially originated. Moreover, based on the data obtained in this study, no correlation can be observed considering the cultivar or the matrix from which the samples were isolated. However, further investigations covering a more extended sample area, a wider temporal distribution and sampling a higher number of isolates, may contribute to clarify whether species, geographical areas and cultivars are correlated.

The study also highlighted a high genetic variation between the two most abundant species, *C*. *godetiae* and *C*. *fioriniae*. Particularly, *C*. *godetiae* presented in seven distinct haplotypes while *C*. *fioriniae* resulted in four haplotypes, although a higher number of samples were obtained during the study. Proportionally, the number of haplotypes over the number of isolates resulted similar in both species, with isolates differing from each other for only one to seventeen nucleotide variations.

Interestingly, one isolate of *C*. *godetiae* was isolated from an insect body (2015-4-1). A scale insect, which did not present any symptom of disease, alive at the time of sampling, was caught and assessed for the presence of *Colletotrichum* sp. The insect was sampled because in 2010, one year before the epidemic event occurred, some areas suffered a big attack of cochineals. Although the capacity of this *C*. *godetiae* isolate to cause disease on the insect was not investigated, the ability of this fungus to colonize and infect insects is documented^19,20^. Similarly, Gaffuri *et al*. 2015^21^ reported the presence of *Colletotrichum acutatum sensu lato* on the Asian chestnut gall wasp (*Dryocosmus kuriphilus*) affecting chestnut (*Castanea sativa*); authors speculate about the ecological role of the insect in the spread of this fungus on other chestnut plants. Undoubtedly, the presence of *C*. *godetiae* on the body of the insect should be investigated considering the ability of the insect to act as a pathogen vector, especially because adult male insects are winged and able to fly and certain stadia of the nymph, called crawlers, are able to move and are considered the main dispersal agents for Coccoidea^22^. Scale insects are also a considerable inoculum source, since female insects heavily feed on different parts of the plant causing important injuries on the tissues, thus facilitating the pathogen penetration^23^.

Pathogenicity tests revealed that two isolates of *C*. *godetiae* (2015-39-2 and 2015-19-1), one of the most abundant species isolated from walnuts affected by anthracnose, produced smaller lesions compared to the other strains when artificially inoculated on fruit. Similar situations have been reported in other pathosystems; for example *C*. *gloeosporioides* species are found only occasionally on strawberry in the UK, though *in vitro* assays reported those as the most aggressive species^24^. The large presence of *C*. *godetiae* on anthracnose lesions may be related to environmental factors, which promote the pathogen diffusion causing a population burst. Further studies, using a more consistent set of isolates and cultivars, are needed to obtain additional data about the aggressiveness of the isolates and the susceptibility of the tested cultivars to *Colletotrichum* spp.

Characterization of the *Colletotrichum* species associated with walnut anthracnose provides considerable knowledge and allows targeted treatments to be implemented. This is of particular concern considering that distinct *Colletotrichum* species respond differently to specific groups of chemical compounds^25,26^. Moreover, the knowledge of the etiological agents of a disease allows the development of diagnostic procedures that can help to monitor and limit the disease. Finally, in order to better elucidate the epidemiology and the pathogen behaviour, it is important to define those factors contributing to species abundance.

## Material and Methods

### Sampling

#### Plant tissues for metabarcoding analysis

Walnut buds were collected from 17 parcels during May-June 2016. In total, 10 parcels were surveyed in South-East (SE) of France (Two parcels in: Beaulieu, 38470; Cras, 38210. And one parcel in: Laissaud, 73800; La Buissière, 38530; Geyssans, 26750; Saint Romans, 38160; Peyrins, 26380; La Motte, 26470) and 7 in South-West (SW) of France (One parcel in: Toulenne, 33210; Terrasson La Villedieu, 24120; Puybrun, 46130; Saint Cybranet, 24250; and three parcels in: Montvalent, 46600) (Fig. 3).

For each parcel, twenty walnut buds from 10 different plants were cut with a sterilized scalpel, mixed and ground with liquid nitrogen in an autoclaved mortar and pestle. DNA was extracted from plant tissues using FastDNA^®^ SPIN kit (MP Biomedicals, Santa Ana, CA, USA) following the manufacturer’s instructions. Quality and concentration of purified DNA were determined using a UV spectrophotometer (NanoDrop1000, Thermo Scientific, USA), and dilutions of at least 10 ng/μL were prepared for each DNA sample using nuclease-free water (Promega, Madison, WI, USA).

#### *Colletotrichum* spp. isolation and morphological description

From July 2015 to May 2016, plant tissue samples were collected from 36 parcels in 16 locations of southern France as shown in Fig. 3. Isolation was performed on fruits, buds, leaves and stems of walnut trees affected by walnut anthracnose.

Collected plant material was cut in small pieces, washed three times (the first one by using a 1% (v/v) NaClO water solution for 1 min, then twice for 2 min using sterile water) and dried on a paper sheet in sterile conditions. Samples were placed in Petri dishes (90 mm) containing Potato Dextrose Agar medium (PDA, Difco Laboratories, USA) and 100 ppm of streptomycin sulphate (Sigma-Aldrich, St Louis, MO, USA), then incubated for at least four days at room temperature. After four/seven days, three to five small agar plugs containing fungal mycelium, identified as *Colletotrichum* sp. by macroscopic and microscopic observations, were transferred to a fresh PDA plate and incubated in the dark at 25 °C for 10 days. One sample (2015-4-1) was obtained from an asymptomatic insect (Hemiptera: Sternorrhyncha: Coccidae) isolated from the branch of a walnut tree.

Cultures were maintained at 25 °C on PDA for up to a week under a 12 h light/dark cycle. Long-term storage involved cryoconservation of spores in liquid nitrogen.

Morphological observations (mycelium colour, texture, zonation, growing margin, and colour of the reverse side) of all isolates were made on cultures grown on PDA plates incubated at room temperature (~20 °C) under natural daylight^27^.

Observations and measurements of conidial size and shape have been made by microscopic observation at ×1000 on spores (20 randomly chosen) harvested after 10 to 14 days incubation and mounted in cotton blue^27^.

### Metabarcoding analysis of *Colletotrichum* spp. diversity in walnut buds

A total of 17 samples were used for amplicon PCRs and Illumina Miseq PE300 sequencing, which was performed at the McGill University and Génome Québec Innovation Centre, Montréal, Canada. Primers ITS1F and ITS4^28^ were used to amplify the internal transcribed spacer.

#### Data Analysis and Statistics

Although expected, a low level of joined pair reads for the analysis of ITS sequences were obtained, leading us to choose an alternative approach with QIIME^29^. The forward and reversed reads were merged in both multiple fasta files independently, using *multiple_split_libraries_fastq*.*py*.

ITS1 and ITS2 regions were first extracted separately from read1 and read2 nonchimera-fasta files respectively, using ITSx^30^ before being concatenated in a new fasta file. Chimera detection was made in the new fasta file, with ITS1 and ITS2 concatenated and lacking in 5.8 region sequence, using the UCHIME algorithm^31^ with vsearch v1.1.3 (https://github.com/torognes/vsearch) and the UNITE/INSDC representative/reference sequences version 7.0^32^ as reference database. Only non-chimeric sequences were used for OTU picking using the QIIME script *pick_open_reference_otus*.*py*, with BLAST^33^ as taxonomic assignment method and a modified database from UNITE plus INSD non-redundant ITS database version 7.1^34^. The modified database was obtained by extracting, using ITSx software, and concatenating ITS1 and ITS2 region sequences from UNITE v7.1 database. To minimize the overestimation of rare OTUs in the community analysis, we include only OTUs with sequence count greater than 10^35,36^. OTUs with “No blast hit” were also discarded to determine the total number of ITS sequences obtained per sample.

For taxonomic assignment at *Colletotrichum* species complex level, the same approach and parameters were used for OTU selection with a home-made ITS-*Colletotrichum* database. The database was obtained selecting entire ITS sequences from representative strains according to currently accepted species of *Colletotrichum*^37^. Species were selected based on phylogenetic distribution in order to cover the diversity of the genus. ITS1 and ITS2 region sequences were extracted using ITSx software, and concatenated. Only OTUs with e-value = 0 and 97% of similarity based on blastn results against ITS-*Colletotrichum* database were selected. All the ITS raw reads files have been deposited at NCBI and are available under Bioproject ID SRP126756, with the BioSample accession numbers from SRS2758044 to SRS2758060.

### Multi-locus phylogenetic analysis of *Colletotrichum* species associated with walnut anthracnose

#### Genomic DNA extraction and PCR amplification

10-day-old fungal mycelium was scraped from the surface of a PDA plate using a sterile scalpel and transferred into a sterile 2 mL tube. Genomic DNA was then extracted using the FastDNA® SPIN kit (MP Biomedicals, Santa Ana, CA, USA) following the manufacturer’s instructions with an initial homogenization step using the Retsch MM400 instrument (Retsch GmbH) at 30 Hz for 30 sec, for two times. The DNA was resuspended in 100 µL of sterile nuclease-free water, quantified and checked in quality using a NanoDrop ND-1000 spectrophotometer (Thermo Scientific, DE, USA). DNA aliquots were stored at a temperature of −20 °C for further use.

In order to establish the species complex designation, for each isolate, the internal transcribed spacer (ITS) region, partial sequence of the glyceraldehyde-3-phosphate dehydrogenase (GAPDH) gene and partial sequence of the beta-tubulin 2 gene (TUB2) (exons 3 through 6, including introns 2 through 4), regions were initially sequenced and compared with reference sequences^38^. Other loci were subsequently amplified to determine the species designation according to Damm *et al*.^10^ for the *C*. *acutatum* species complex and to Weir *et al*.^16^ for the *C*. *gloeosporioides* species complex.

For isolates belonging to the *C*. *acutatum* species complex, partial sequences of the chitin synthase 1 gene (CHS-1), actin gene (ACT) and histone H3 gene (HIS3) were amplified and sequenced. For isolates identified as belonging to the *C*. *gloeosporioides* species complex, partial sequence of the chitin synthase 1 gene (CHS-1), actin gene (ACT), glutamine synthetase (GS), calmodulin (CAL) and Apn2/Mat1-2-1 intergenic spacer (ApMAT) were amplified and sequenced.

Amplification reactions were performed in 25 μL volume using 0.025 U/μL of GoTaq Flexi DNA polymerase (Promega) and 1 × GoTaq Flexi buffer (Promega), 25–50 ng of template DNA, 0.08 μM of each primer, 2 mM of MgCl_2_ and 0.2 mM of 10 mM dNTP mix (Promega). For GAPDH and TUB2 genes, primer concentration was increased to 0.2 μM while dNTP mix concentration was decreased to 0.08 mM. A list of the primers and conditions used in this study is reported in Table 3.

**Table 3:** List of primers and PCR conditions used in this study.

Loci	Primer names	Sequences (5′-3′)	PCR conditions used
ITS^46^	ITS5	GGA AGT AAA AGT CGT AAC AAG G	5′ at 95 °C, 30 × (1′ at 95 °C, 1′ at 55 °C, 1′ at 72 °C), 10′ at 72 °C
	ITS4	TCC TCC GCT TAT TGA TAT GC	
GAPDH^47^	GDF1	GCC GTC AAC GAC CCC TTC ATT GA	5′ at 95 °C, 35 × (30″ at 95 °C, 30″ at 60 °C, 30″ at 72 °C), 7′ at 72 °C
	GDR1	GGG TGG AGT CGT ACT TGA GCA TGT	
TUB2^48^	BT2Fd	GTB CAC CTY CAR ACC GGY CAR TG	2′ at 95 °C, 30 × (1′ at 95 °C, 1′ at 67 °C, 1′ at 72 °C), 5′ at 72 °C
	BT4R	CCR GAY TGR CCR AAR ACR AAG TTG TC	
CHS-1*^49^	CHS-79F	TGG GGC AAG GAT GCC TGG AAG AAG	2′ at 95 °C, 40 × (1′ at 95 °C, 30″ at 62 °C, 20″ at 72 °C), 5′ at 72 °C
	CHS-354R	TGG AAG AAC CAT CTG TGG GAG TTG	
ACT*^49^	ACT-512F	ATG TGC AAG GCC GGT TTC GC	2′ at 95 °C, 40 × (1′ at 95 °C, 30″ at 57 °C, 25″ at 72 °C), 5′ at 72 °C
	ACT-783R	TAG GAG TCC TTC TGA CCC AT	
HIS3*^50^	CYLH3Fext	AGT CCA CTG GTG GCA AGG C	2′ at 95 °C, 40 × (1′ at 95 °C, 30″ at 57 °C, 25″ at 72 °C), 5′ at 72 °C
	CYLH3R	AGC TGG ATG TCC TTG GAC TG	
GS^16^	GSF3	TCG CCC GCA CTG CTG CAG CCGG	4′ at 95 °C, 40 × (30″ at 95 °C, 30″ at 55 °C, 45″ at 72 °C), 7′ at 72 °C
	GSR2	GAA CCG TCG AAG TTC CAC	
CAL*^16^	CL1C	GAA TTC AAG GAG GCC TTC TC	4′ at 95 °C, 40 × (30″ at 95 °C, 30″ at 55 °C, 45″ at 72 °C), 7′ at 72 °C
	CL2C	TTC TGC ATC ATG AGC TGG AC	
ApMAT^51^	AM-F	TCA TTC TAC GTA TGT GCC CG	5′ at 95 °C, 40 × (45″ at 95 °C, 45″ at 62 °C, 1′ at 72 °C), 7′ at 72 °C
	AM-R	CCA GAA ATA CAC CGA ACT TGC	

*primers modified on the basis of *Colletotrichum* spp. sequences available.

Amplification products were analysed by electrophoresis in 1 × TAE buffer (40 mM Tris-acetate, 1 mM EDTA) with 1% (w/v) agarose gel (LE, analytical grade agarose; Promega) prepared using 1 × TAE buffer and detected by UV fluorescence after GelRed™ (Biotium Inc., CA) staining, according to manufacturer’s instructions. The BenchTop 100-bp DNA ladder (Promega) was used as molecular size marker. PCR products were sent to Eurofins MWG (Ebersberg, Germany) for purification and sequencing in forward and reverse, using the same primers used for PCR. ABI trace files were analysed and consensus sequences were generated using Geneious^®^ 10.0.6 (Biomatters, http://www.geneious.com).

#### Phylogenetic analysis and species identification

To establish the species complex of each isolate, a phylogenetic tree of the *Colletotrichum* genus was constructed using a concatenated alignment of ITS, TUB2 and GAPDH^39^. For the isolates belonging to the *acutatum* complex, phylogenetic analysis was conducted using a sequence dataset enriched with 39 ex-type and other reference strains of species belonging to the *C*. *acutatum* complex, *C*. *orchidophilum* was used as outgroup. For the isolate belonging to the *gloeosporioides* complex, sequences of 39 reference strains were used and *C*. *sydowii* was used as outgroup. All reference sequences based on Marin-Felix *et al*.^38^ are available and listed in Supplementary Table 2.

The sequences obtained were aligned using MAFFT v. 7.304^40^. Multiple sequence alignments were exported to MEGA7^41^ and the best-fit substitution model was calculated for each separate sequence dataset. The multi-locus concatenated alignment was performed using Geneious 10.0.6. Using MrBayes 3.2.6^42^, the Markov chain Monte Carlo (MCMC) algorithm was performed to generate phylogenetic trees with Bayesian posterior probabilities for combined sequence datasets using, for each locus, the nucleotide substitution models determined by MEGA7. Four MCMC chains were run simultaneously for random trees for 5,000,000 generations. Samples were taken every 1,000 generations. The first 25% of trees were discarded as burn-in phase of each analysis and posterior probabilities were determined from the remaining trees.

To visualize intraspecific evolutionary and geographic relationships between isolates the Median-joining network algorithm^43^ was used to build a haplotypes network using the software PopART v1.7^44^. Analysis of molecular variance (AMOVA) was performed with Arlequin 3.5^45^ to compare the genetic structure of 2 groups: samples from South East (SE; haplotypes = 6, isolates = 31), samples from South West (SW; haplotypes = 10, isolates = 83). For this purpose, conventional F-statistics and 10,000 permutations to test significance were used with haplotype frequencies.

### Pathogenicity tests

Eight representative *Colletotrichum* strains (*C*. *godetiae* 2015-19-1, 2015-24-3 and 2015-39-2; *C*. *fioriniae* 2015-19-2, 2015-26-1 and 2015-41-1; *C*. *nymphaeae* 2016-5-1; *C*. *gloeosporioides sensu lato* 2016-1-3; Table 1), selected among the isolates obtained during this study, were used to perform pathogenicity tests on artificially wounded fruits (cultivar Lara).

Fruits, harvested 100 days after the beginning of fruit enlargement, were first washed with distilled water and then surface sterilized using a 70% (v/v) ethanol solution for 1 min, rinsed twice with distilled water and dried on a paper sheet. Surface sterilized fruits were wounded on the pericarp using a 2 mL pipette tip and an agar plug (0.2 cm in diameter) containing the fungal mycelium, was placed in the wound. 5 Wounded fruits inoculated with agar without mycelium were used as control. For each strain 5 fruits were inoculated. The test was independently replicated twice. Inoculated fruits were then incubated in a moist chamber at 24 °C.

The development of the necrosis was daily monitored and the two perpendicular necrosis diameters were recorded 4, 8 and 14 days after the first symptoms appeared, corresponding to 9, 13 and 19 days post inoculation. Data from the final measurements were submitted to analysis of variance (ANOVA and Tukey’s multiple post hoc range test), with isolate as independent variable, by using Systat 11 (Systat Software, USA) and assuming P < 0.05 as significant level.

At the end of the experiment, each strain was re-isolated from the affected fruits and cultured on PDA and streptomycin sulphate in order to confirm the identity (based on morphological characters) of the causal agent.

## Electronic supplementary material

Supplementary information
