# Supplementary material for: Combined Metabarcoding and Multi-locus approach for Genetic characterization of Colletotrichum species associated with common walnut (Juglans regia) anthracnose in France
Source: Sci Rep. 2018 Jul 17;8:10765. doi: 10.1038/s41598-018-29027-z (PMC6050315; doi:10.1038/s41598-018-29027-z)
Supplement: Supplementary file 1 — Supplementary information [file 41598_2018_29027_MOESM1_ESM.pdf]

Combined Metabarcoding and Multi-locus approach for Genetic characterization of *Colletotrichum* species associated with common walnut (*Juglans regia*) anthracnose in France.

Daniele Da Lio[1,#], José F. Cobo-Díaz[1], Cyrielle Masson[2], Morgane Chalopin[1], Djiby Kebe[2], Michel Giraud[3], Agnes Verhaeghe[4], Patrice Nodet[1], Sabrina Sarrocco[5], Gaetan Le Floch[1], Riccardo Baroncelli[1,*].

[1] Laboratoire Universitaire de Biodiversité et Ecologie Microbienne, IBSAM, ESIAB, Université de Brest, EA 3882, Technopôle Brest-Iroise, 29280 Plouzané, France.

[2] Station Expérimentale Nucicole Rhône-Alpes, 385 A Route de St Marcellin, 38160 Chatte, France

[3] Centre Technique Interprofessionnel des Fruits et Légumes, Centre de Lanxade, 28 route des Nébouts, 24130 Prigonrieux, France

[4] CTIFL/SENuRA, 385 A Route de St Marcellin, 38160 Chatte, France

[5] Dipartimento di Scienze Agrarie, Alimentari e Agro-ambientali, Università di Pisa, Via del Borghetto 80 – 56124 - Pisa, Italy

[#] present address: Dipartimento di Scienze Agrarie, Alimentari e Agro-ambientali, Università di Pisa, Via del Borghetto 80 – 56124 - Pisa, Italy

* Corresponding author: **Riccardo Baroncelli** (riccardobaroncelli@gmail.com)

Supplementary Table 1. Information about the final alignments used for phylogenetic analyses

Locus	boundaries	conserved	parsimony info	singleton	best substitution model
Colletotrichum acutatum species complex					
ITS	1-500	454	21	20	K2P+G
CHS-1	501-763	224	24	15	K2P+G
TUB2	764-1255	362	69	60	K2P+G
ACT	1256-1496	159	43	38	K2P+G
HIS3	1497-1875	274	82	22	TN93+G
GAPDH	1876-2124	118	64	53	K2P+G
Concatenated	1-2124	1591	303	208	-
Colletotrichum gloeosporioides species complex					
ITS	1-615	523	21	61	K2P+G
CHS-1	616-916	231	35	35	K2P+G
CAL	917-1705	590	95	93	K2P+G
ACT	1706-2006	162	38	84	K2P
SOD2	2007-2380	278	51	45	K2P+G
TUB2	2381-3363	666	115	160	K2P+G
GS	3364-4369	590	122	204	T92+G
GAPDH	4370-4678	113	82	93	K2P
ApMAT	4679-5716	505	209	276	K2P
Concatenated	1-5716	3658	768	1051	-

Supplementary Table 2. Reference sequences used in the phylogenetic analyses.

Species	strain	ITS	GAPDH	TUB2	CHS-1	ACT	HIS3	GS	CAL	ApMAT
C. abscissum	CBS 134727	KC204988	KC205022	KC205056	KC205039	KC205073	KC205003	-	-	-
C. acerbum	CBS 128530	JQ948459	JQ948790	JQ950110	JQ949120	JQ949780	JQ949450	-	-	-
C. acutatum	CBS 112996	JQ005776	JQ948677	JQ005860	JQ005797	JQ005839	JQ005818	-	-	-
C. australe	CBS 116478	JQ948455	JQ948786	JQ950106	JQ949116	JQ949776	JQ949446	-	-	-
C. brisbanense	CBS 292.67	JQ948291	JQ948621	JQ949942	JQ948952	JQ949612	JQ949282	-	-	-
C. cairnsense	CBS 140847	KU923672	KU923704	KU923688	KU923710	KU923716	KU923722	-	-	-
C. carthami	MAFF 239358	•	•	•	•	•	•	-	-	-
C. chrysanthemi	CBS 126518	JQ948271	JQ948601	JQ949922	JQ948932	JQ949592	JQ949262	-	-	-
C. citri	ZJUC41	KC293581	KC293741	KC293661		KC293621		-	-	-
C. cosmi	CBS 853.73	JQ948274	JQ948604	JQ949925	JQ948935	JQ949595	JQ949265	-	-	-
C. costaricense	CBS 330.75	JQ948180	JQ948510	JQ949831	JQ948841	JQ949501	JQ949171	-	-	-
C. cuscutae	IMI 304802	JQ948195	JQ948525	JQ949846	JQ948856	JQ949516	JQ949186	-	-	-
C. fioriniae	IMI 504882	+	+	+	+	+	+	-	-	-
C. godetiae	CBS 133.44	JQ948402	JQ948733	JQ950053	JQ949063	JQ949723	JQ949393	-	-	-
C. guajavae	IMI 350839	JQ948270	JQ948600	JQ949921	JQ948931	JQ949591	JQ949261	-	-	-
C. indonesiense	CBS 127551	JQ948288	JQ948618	JQ949939	JQ948949	JQ949609	JQ949279	-	-	-
C. johnstonii	IMI 357027	JQ948443	JQ948774	JQ950094	JQ949104	JQ949764	JQ949434	-	-	-
C. kinghornii	CBS 198.35	JQ948454	JQ948785	JQ950105	JQ949115	JQ949775	JQ949445	-	-	-
C. laticiphilum	CBS 112989	JQ948289	JQ948619	JQ949940	JQ948950	JQ949610	JQ949280	-	-	-
C. limetticola	CBS 114.14	JQ948193	JQ948523	JQ949844	JQ948854	JQ949514	JQ949184	-	-	-
C. lupini	CBS 109225	JQ948155	JQ948485	JQ949806	JQ948816	JQ949476	JQ949146	-	-	-
C. melonis	CBS 159.84	JQ948194	JQ948524	JQ949845	JQ948855	JQ949515	JQ949185	-	-	-
C. nymphaeae	IMI 504889	+	+	+	+	+	+	-	-	-
C. paranaense	IMI 384185	JQ948191	JQ948521	JQ949842	JQ948852	JQ949512	JQ949182	-	-	-
C. paxtonii	IMI 165753	JQ948285	JQ948615	JQ949936	JQ948946	JQ949606	JQ949276	-	-	-
C. phormii	CBS 118194	JQ948446	JQ948777	JQ950097	JQ949107	JQ949767	JQ949437	-	-	-
C. pyricola	CBS 128531	JQ948445	JQ948776	JQ950096	JQ949106	JQ949766	JQ949436	-	-	-
C. rhombiforme	CBS 129953	JQ948457	JQ948788	JQ950108	JQ949118	JQ949778	JQ949448	-	-	-
C. salicis	CBS 607.94	+	+	+	+	+	+	-	-	-
C. scovillei	CBS 126529	JQ948267	JQ948597	JQ949918	JQ948928	JQ949588	JQ949258	-	-	-
C. simmondsii	CBS 122122	+	+	+	+	+	+	-	-	-
C. sloanei	IMI 364297	JQ948287	JQ948617	JQ949938	JQ948948	JQ949608	JQ949278	-	-	-
C. tamarilloi	CBS 129814	JQ948184	JQ948514	JQ949835	JQ948845	JQ949505	JQ949175	-	-	-
C. walleri	CBS 125472	JQ948275	JQ948605	JQ949926	JQ948936	JQ949596	JQ949266	-	-	-
Colletotrichum sp.	CBS 101611	JQ948196	JQ948526	JQ949847	JQ948857	JQ949517	JQ949187	-	-	-
Colletotrichum sp.	CBS 129810	JQ948179	JQ948509	JQ949830	JQ948840	JQ949500	JQ949170	-	-	-

Species	strain	ITS	GAPDH	TUB2	CHS-1	ACT	HIS3	GS	CAL	ApMAT
Colletotrichum sp.	CBS 129820	JQ948183	JQ948513	JQ949834	JQ948844	JQ949504	JQ949174	-	-	-
Colletotrichum sp.	CBS 129821	JQ948182	JQ948512	JQ949833	JQ948843	JQ949503	JQ949173	-	-	-
Colletotrichum sp.	CBS 129823	JQ948192	JQ948522	JQ949843	JQ948853	JQ949513	JQ949183	-	-	-
C. orchidophilum	CBS 632.80	JQ948152	JQ948482	JQ949803	JQ948813	JQ949473	JQ949143	-	-	-
C. aenigma	ICMP 18608	JX010244	JX010044	JX010389	JX009774	JX009443	-	JX010078	JX009683	KM360143
C. aeschynomenes	ICMP 17673	JX010176	JX009930	JX010392	JX009799	JX009483	-	JX010081	JX009721	–
C. alatae	ICMP 17919	JX010190	JX009990	JX010383	JX009837	JX009471	-	JX010065	JX009738	KC888932
C. alienum	ICMP 12071	JX010251	JX010028	JX010411	JX009882	JX009572	-	JX010101	JX009654	KM360144
C. aotearoa	ICMP 18537	JX010205	JX010005	JX010420	JX009853	JX009564	-	JX010113	JX009611	KC888930
C. asianum	ICMP 18580	FJ972612	JX010053	JX010406	JX009867	JX009584	-	JX010096	FJ917506	FR718814
C. camelliae	CGMCC 3.14925	KJ955081	KJ954782	KJ955230	-	KJ954363	-	KJ954932	KJ954634	KJ954497
C. changpingense	MFLUCC 15-0022	KP683152	KP852469	KP852490	KP852449	KP683093	-	-	-	-
C. clidemiae	ICMP 18658	JX010265	JX009989	JX010438	JX009877	JX009537	-	JX010129	JX009645	KC888929
C. conoides	CGMCC 3.17615	KP890168	KP890162	KP890174	KP890156	KP890144	-	-	KP890150	-
C. cordylinicola	ICMP 18579	JX010226	JX009975	JX010440	JX009864	HM470235	-	JX010122	HM470238	JQ899274
C. endophytica	MFLUCC 13–0418	KC633854	KC832854	-	-	KF306258	-	-	KC810018	-
C. fructicola	ICMP 18581	JX010165	JX010033	JX010405	JX009866	FJ907426	-	JX010095	FJ917508	JQ807838
C. fructivorum	CBS 133125	JX145145	-	JX145196	-	-	-	-	-	JX145300
C. gloeosporioides	CBS 112999	JQ005152	JQ005239	JQ005587	JQ005326	JQ005500	-	JX010085	JQ005673	JQ807843
C. grevilleae	CBS 132879	KC297078	KC297010	KC297102	KC296987	KC296941	-	KC297033	KC296963	-
C. grossum	CGMCC3.17614	KP890165	KP890159	KP890171	KP890153	KP890141	-	-	KP890147	-
C. hebeiense	MFLUCC13–0726	KF156863	KF377495	KF288975	KF289008	KF377532	-	-	-	-
C. henanense	CGMCC 3.17354	KJ955109	KJ954810	KJ955257	-	KM023257	-	KJ954960	KJ954662	KJ954524
C. horii	ICMP 10492	GQ329690	GQ329681	JX010450	JX009752	JX009438	-	JX010137	JX009604	JQ807840
C. jiangxiense	CGMCC 3.17363	KJ955201	KJ954902	KJ955348	-	KJ954471	-	KJ955051	KJ954752	KJ954607
C. kahawae subsp. ciggaro	ICMP 18539	JX010230	JX009966	JX010434	JX009800	JX009523	-	JX010132	JX009635	-
C. kahawae subsp. kahawae	ICMP 17816	JX010231	JX010012	JX010444	JX009813	JX009452	-	JX010130	JX009642	JQ894579
C. musae	ICMP 19119	JX010146	JX010050	HQ596280	JX009896	JX009433	-	JX010103	JX009742	KC888926
C. nupharicola	ICMP 18187	JX010187	JX009972	JX010398	JX009835	JX009437	-	JX010088	JX009663	JX145319
C. proteae	CBS 132882	KC297079	KC297009	KC297101	KC296986	KC296940	-	-	KC296960	-
C. psidii	ICMP 19120	JX010219	JX009967	JX010443	JX009901	JX009515	-	JX010133	JX009743	KC888931
C. queenslandicum	ICMP 1778	JX010276	JX009934	JX010414	JX009899	JX009447	-	JX010104	JX009691	KC888928
C. rhexiae	CBS 133134	JX145128	-	JX145179	-	-	-	-	-	JX145290
C. salsolae	ICMP 19051	JX010242	JX009916	JX010403	JX009863	JX009562	-	JX010093	JX009696	KC888925
C. siamense	ICMP 18578	JX010171	JX009924	JX010404	JX009865	FJ907423	-	JX010094	FJ917505	JQ899289
C. syzygicola	MFLUCC 10–0624	KF242094	KF242156	KF254880	-	KF157801	-	KF242125	KF254859	-
C. temperatum	CBS 133122	JX145159	-	JX145211	-	-	-	-	-	JX145298
C. theobromicola	ICMP 18649	JX010294	JX010006	JX010447	JX009869	JX009444	-	JX010139	JX009591	KC790726
C. ti	ICMP 4832	JX010269	JX009952	JX010442	JX009898	JX009520	-	JX010123	JX009649	KM360146

Species	strain	ITS	GAPDH	TUB2	CHS-1	ACT	HIS3	GS	CAL	ApMAT
C. tropicale	ICMP 18653	JX010264	JX010007	JX010407	JX009870	JX009489	-	JX010097	JX009719	KC790728
C. viniferum	GZAAS 5.08601	JN412804	JN412798	JN412813	-	JN412795	-	JN412787	JQ309639	-
C. wuxiense	CGMCC 3.17894	KU251591	KU252045	KU252200	KU251939	KU251672	-	KU252101	KU251833	KU251722
C. xanthorrhoeae	ICMP 17903	JX010261	JX009927	JX010448	JX009823	JX009478	-	JX010138	JX009653	KC790689
C. sydowii	CBS 135819	KY263783	KY263785	KY263793	KY263787	KY263791	-	-	-	-

• = Sequences retrieved directly from the genome deposited on NARO database (http://www.gene.affrc.go.jp/)
+ = Sequences retrieved directly from the genome deposited on NCBI database (http://www.ncbi.nlm.nih.gov/)
